# Berberine Inhibits the Expression of SCT through miR-214-3p Stimulation in Breast Cancer Cells

**DOI:** 10.1155/2020/2817147

**Published:** 2020-11-29

**Authors:** Congyuan Zhu, Jianping Li, Yuming Hua, Jingli Wang, Ke Wang, Jingqiu Sun

**Affiliations:** Department of General Surgery, The Affiliated Hospital of Jiangnan University, Wuxi Third People's Hospital, Wuxi, Jiangsu 214002, China

## Abstract

In this study, we aimed to evaluate the suppressive abilities of berberine (BBR) on MCF-7 and MDA-MB-231 cells and confirm its underlying mechanisms on miR-214-3p. We first built a panel of 18 miRNAs and 9 lncRNAs that were reported to participate in the mechanism of breast cancer. The RT-qPCR results suggested that BBR illustrated a dosage-dependent pattern in the stimulation to miR-214-3p in both MCF-7 and MDA-MB-231 cells. Then, we performed gain-and-lose function tests to validate the role of miR-214-3p contributing to the anticancer effects of BBR. Both BBR and miR-214-3p mimic reduced the cell viability, repressed migration and invasion capacities, increased rates of total apoptotic cells and ratio of Bax/Bcl-2, and increased the percentage of G2/M cells of MCF-7 and MDA-MB-231 cells by colony formation and CKK8 assay, scratch wound healing and gelatin-based 3D conformation assay, transwell invasion assay, and cell cycle analysis, respectively. However, miR-214-3p inhibitor counteracted all these effects of BBR. Based on the bioinformatics analysis and dual-luciferase reporter test, we identified binding sites between SCT and miR-214-3p. We further confirmed that BBR massively and dose-dependently reduced the mRNA expression and protein levels of SCT in both MCF-7 and MDA-231 cells. We testified that both miR-214-3p mimic and BBR could decrease the mRNA expression and protein levels of SCT, while miR-214-3p inhibitor weakened these reductions. In conclusion, BBR suppressed MCF-7 and MDA-MB-231 breast cancer cells by upregulating miR-214-3p and increasing its inhibition to SCT.

## 1. Introduction

Based on the results of epidemiological investigation, it has been reported that breast cancer is becoming one of the major types of cancer and contributes to the highest mortality rate in gynecologic malignancies [1]. Currently, the mainstream treatments of breast cancer are regional avenues, including radiation and surgery and general tools like chemotherapy and biologic therapies [2]. In order to improve the management of breast cancer, it is necessary to exploit novel therapeutic target.

Noncoding RNA, including microRNA (miRNA) and long noncoding RNA (lncRNA), participate in various biological processed [3]. Both miRNAs and lncRNAs related to human cancers are referred to as “oncomirs” [4], which are identified as two types: (i) miRNAs or lncRNAs called oncogenes are upregulated or amplified in cancer; (ii) miRNAs or lncRNAs called suppressors are downregulated or deleted in cancer [5]. A handful of miRNAs and lncRNAs, such as miR-101 [6], miR-21 [7], miR-155 [8], LncRNA-H19 [9], lncRNA-SNHG6 [10], and lncRNA-TALNEC2 [11], are considered to participate in the mechanism of proliferation, invasion, apoptosis, and molecular signaling in breast cancer. Therefore, these small regulatory miRNAs work as novel targets for anticancer therapeutic strategies.

The chemical name of berberine (BBR) is 2,3-methylenedioxy-9,10-dimenthoxyprotoberberine chloride. BBR is a kind of isoquinoline alkaloid that is extracted from Coptidis Rhizoma or Huanglian. BBR has been proved to possess numerous protective properties, like antimicrobial, cardioprotective, and antidiabetic activities [12, 13]. Among these properties, anticancer activity of BBR has been widely accepted. BBR shows anticancer activity in plenty of cancers, including breast cancers [14]. It is believed that BBR moderates mitochondria and pathway of caspase to induce the apoptosis of breast cancer cells [15]. However, how miRNA regulation plays a role in the inhibition of breast cancer cells by BBR to is still under ambiguous.

In this study, we first made a panel of 18 miRNAs [6–8, 16–30] and 9 lncRNAs [9, 10, 15, 29–34] that were previously reported to be related with breast cancer. Then, based on a series of tests, we identified miR-214-3p as the crucial miRNA mediating in the antitumor effects of BBR in MCF-7 and MDA-MB-231 breast cancer cells. Furthermore, according to target scan software and a series of tests, we ensured that BBR promoted miR-214-3p expression and suppressed the protein expression of its targets secretin (SCT).

## 2. Materials and Methods

### 2.1. Cell Culture

Two human breast cancer cell lines (MCF-7 and MDA-MB-231) were purchased from Chinese Academy of Sciences cell bank (Shanghai, China). The cells were cultured in RPMI-1640 medium (Gibco, Thermo) supplemented with 10% fetal bovine serum (Gibco, Thermo) and incubated in an atmosphere of 37^o^C humidified and 5% CO_2_.

### 2.2. Introduction of Plasmids and siRNA into Cells

Lipofectamine® 3000 (Thermo Fisher Scientific, Inc.) was used to transfect the plasmids into cells in accordance with the protocols. Reagent of miR-214-3p mimic or miR-214-3p inhibitor (Guangzhou RiboBio Co., Ltd.) was employed to up- or downregulate the levels of miR-214-3p in 6-well plates with a density of 2 × 10^5^ cells in each well. The negative control (NC) was manipulated by a scrambled miRNA.

### 2.3. Cell Proliferation Assay

We used the Cell Counting Kit 8 (CCK8, Beyotime, China) assay and colony formation assay to estimate the effect of BBR on cell proliferation. Briefly, 7 × 10^3^ cells/well were seeded in 96-well plates and treated with BBR (HPLC ≥ 99%, purchased from Meilun Biologics, Dalian China). After 72 h of incubation, a microplate reader was used at a 450 nm optical density to test the viability of each group cells. In colony formation assay, cells were placed into 6-well plates and maintained in media for two weeks and fixed by methanol and stained by 0.1% crystal violet (Sigma, USA) for 20 min.

### 2.4. Cell Migration and Invasion Assay

The activity of cell migration was evaluated by scratch wound healing assay and gelatin-based 3D conformation assay. In scratch wound healing assay, a sterile tip was used to scratch each well to form a thin “wound.” Before adding the serum-free medium, PBS was manipulated to wash the floating cells. At 0 and 12 h after cell recovery, Image-Pro Plus software was manipulated for measuring cell migration distance, and the data were averaged. In gelatin-based 3D conformation assay, fully-formed 3D structures were transferred to 0.1% gelatin-coated plates and treated with BBR or miR-214-3p mimic or miR-214-3p inhibitor. The migration levels were evaluated after 24 and 48 h. The medium containing 2% FBS was employed to reduce influence of cell proliferation in this test. 3D structures were imaged by microscope Nikon Eclipse TS 110 and quantified by ImageJ software. The migration index was calculated by the area of cells migrating outwards the 3D structure.

Invasion activities of MCF-7 and MDA-MB-231 cells were analyzed by using transwell invasion assay. On the upper surface of the membrane, the chambers were coated with Matrigel (1 : 5; 80 *μ*l/well, BD Biosciences). DMEM with 10% FBS was added to the lower chamber. After 24 h incubation, 4% paraformaldehyde was used to fix the upper chambers for 10 min. Then, it was stained by crystal violet. The microscope (Olympus) was used to count the numbers of passed cells.

### 2.5. Apoptosis Assay and Cell Cycle Analysis

The levels of apoptosis were measured by annexin-V and propidium iodide (PI) staining as previously described [35]. Cells were seeded to 6-well plate and treated with DMSO, BBR, miR-214-3p mimic, or BBR with miR-214-3p inhibitor. After 24 h, cells were harvested and stained with annexin-V and propidium iodide for 20 min and then run on a BD FACSCanto II cell analyser (BD Biosciences, USA). At least 10000 single cell events were acquired per sample and analyzed by FlowJo software v10.5.0 (FlowJo, USA).

The methods of cell cycle analysis were performed according to previous report [36]. Cells were seeded to 6-well plate and treated with DMSO, BBR, miR-214-3p mimic, or BBR with miR-214-3p inhibitor. After 24 h, cells were resuspended in ice-cold Dulbecco's phosphate buffered saline (DPBS) with 70% ethanol. Cells were centrifuged at 300 x*g* for 5 min and resuspended in DPBS. After 2 h staining of propidium iodide and bovine pancreas ribonuclease, cells were run in BD FACSCanto II cell analyser (BD Biosciences, USA). 50000 single cell events were captured per sample and analysed by FlowJo software v10.5.0 (FlowJo, USA).

### 2.6. Detection of miRNAs and lncRNAs

We used TRIzol reagent (Invitrogen, CA) to extract total RNA of MCF-7 and MDA-MB-231 cells in each group. SYBR Green PCR kit (Thermo) was used for PCR amplification. Each sample was provided with three repeated holes. Internal reference of GAPDH was manipulated to adjusting and the data of mRNA expression were calculated by the 2^−ΔΔ^Ct method. Primer sequences were shown in Table 1.

### 2.7. Protein Detection of Levels of SCT

The total protein of MCF-7 and MDA-MB-231 cells was extracted with RIPA lysis method. The protein concentration of each well was detected with a BCA method. Protein content was adjusted to 4 *μ*g/*μ*l, 12% SDS-PAGE electrophoresis separation was carried out, and the membrane was transferred to PVDF membranes after ionization. Staining was carried out with Ponceau working solution. The antibodies of GAPDH (Sigma no. 2275-PC-020) and SCT (Sigma no. AF6387-SP) were diluted to 1 : 5000 and 1 : 1000, respectively. The diluents were added to be sealed overnight at 4°C. Quantity One software was employed to analyze the gray value of scanned protein bands. The relative expression was equal to the ratio of target protein gray value and GAPDH gray value.

### 2.8. Prediction of Target Genes

Targetscan7.2 and miRwalk were used to predict downstream target gene of miR-214-3p. Reporter gene plasmids containing wild-type and mutant SCT 3′UTR, psiCHECK-2 plasmids (Promega, C8021, USA), miR-214-3p mimic, and their controls were transfected into MCF-7 and MDA-MB-231 cells for 48 hours and then used. Dual-luciferase reporter gene detection kits were used for operation. Finally, collected cells were detected by chemiluminescence. Three repetitions were designed for each group of experiments, and each experiment was repeated for three times.

### 2.9. Statistical Analysis

The data were analyzed by SPSS 20.0 software and the results were drawn by Graph Prism 8.0. All the data were expressed as mean ± standard deviation (SD ± means). The comparisons of each group were calculated by Student's *t*-test or one-way ANOVA methods. When *p* <  0.05, there were statistical differences.

## 3. Results

### 3.1. BBR Can Obviously Increase the Expression of miR-214-3p in Both MCF-7 and MDA-MB-231 Cells

**Figure 1:**
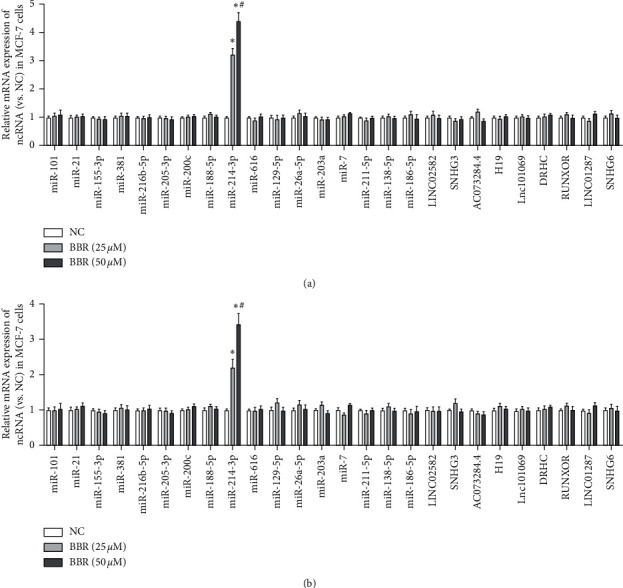
BBR increases the levels of miR-214-3p in both MCF-7 and MDA-MB-231 cells. RT-qPCR results suggested that BBR illustrated a dosage-dependent pattern in the stimulation of miR-214-3p in both MCF-7 and MDA-MB-231 cells in a panel of 18 miRNAs and 9 lncRNAs that were reported to participate in the mechanism of breast cancer. ^*∗*^*p* < 0.05 vs. NC group. ^#^*p* < 0.05 vs. BBR (25um) group.

We first built a panel of 18 miRNAs and 9 lncRNAs that were reported to participate in the mechanism of breast cancer. The RT-qPCR results suggested that BBR illustrated a dosage-dependent pattern in the stimulation to miR-214-3p in both MCF-7 and MDA-MB-231 cells (Figure 1).

### 3.2. BBR Upregulates miR-214-3p Expression to Repress Cell Growth, Invasion, and Migration

**Figure 2:**
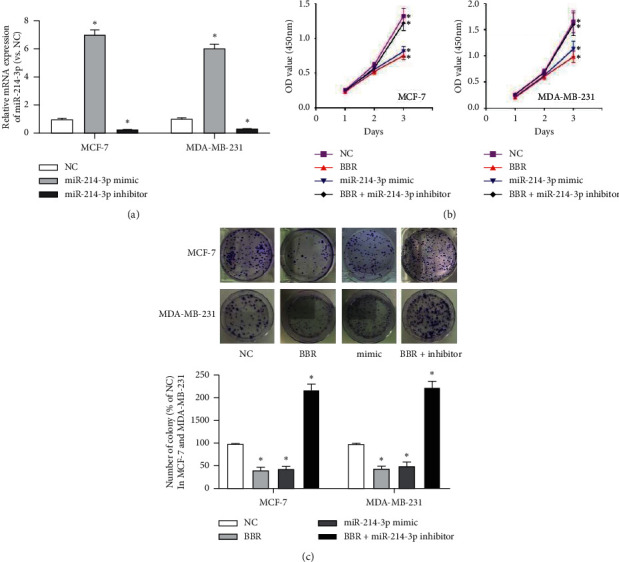
Transfection efficiency and proliferation evaluation. (a) The transfection efficiency was confirmed by RT-qPCR. (b) Colony formation assay demonstrated that both BBR treatment and miR-214-3p mimic could reduce the colony numbers of MCF-7 cells by 56.62% and 60.33% and MDA-MB-231 cells by 50.42% and 56.21%, respectively. (c) CKK8 assay showed that both BBR treatment and miR-214-3p mimic could reduce the cell viability of MCF-7 cells by 35.4% and 27.4%, and MDA-MB-231 cells by 27.4% and 11.5%, respectively. ^*∗*^*p* < 0.05 vs. NC group. ^#^*p* < 0.05 vs. BBR group.

**Figure 3:**
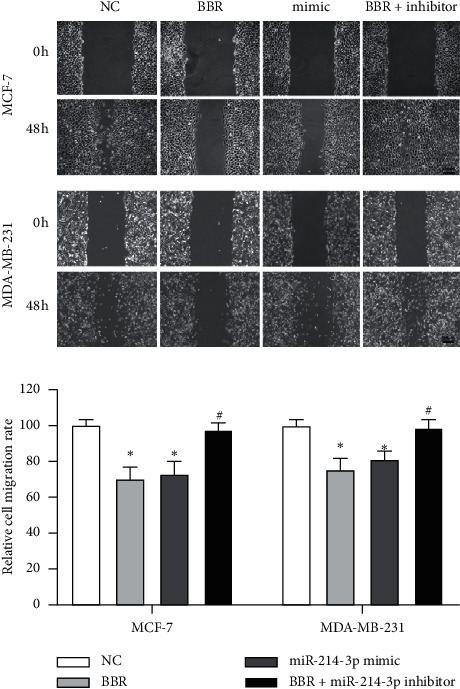
Scratch wound healing assays. MCF-7 and MDA-MB-231 cells were cultured in DMEM 0.1% FBS and treated with 50 *µ*M of BBR, miR-214-3p mimic, or miR-214-3p inhibitor for 48 h. Wound closure was monitored and calculated using the software ImageJ. ^*∗*^*p* < 0.05 vs. NC group. ^#^*p* < 0.05 vs. BBR group.

**Figure 4:**
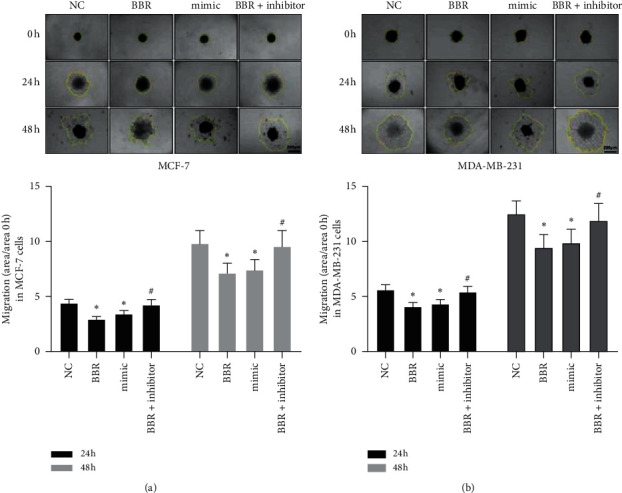
Gelatin-based 3D conformation assays. BBR treatment and miR-214-3p mimic could repress MCF-7 (a) and MDA-MB-231 (b) moving outwards from the 3D structures and being confined to the center region, where they were initially seeded. ^*∗*^*p* < 0.05 vs. NC group. ^#^*p* < 0.05 vs. BBR group.

**Figure 5:**
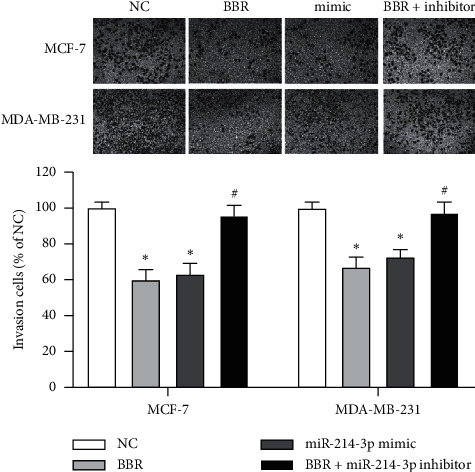
Transwell invasion assays. BBR and miR-214-3p mimic prevented the invasion capacities of MCF-7 and MDA-MB-231 cells. ^*∗*^*p* < 0.05 vs. NC group. ^#^*p* < 0.05 vs. BBR group.

Then, we performed gain-and-lose function tests to validate the role of miR-214-3p contributing to the anticancer effects of BBR on MCF-7 and MDA-MB-231 cells. The transfection efficiency was confirmed by RT-qPCR (Figure 2(a)). The results of colony formation assay demonstrated that both BBR treatment and miR-214-3p mimic could reduce the colony numbers of MCF-7 cells by 56.62% and 60.33% and MDA-MB-231 cells by 50.42% and 56.21%, respectively (Figure 2(b)). CKK8 assay showed that both BBR treatment and miR-214-3p mimic could reduce the cell viability of MCF-7 cells by 35.4% and 27.4% and MDA-MB-231 cells by 27.4% and 11.5%, respectively (Figure 2(c)). These results indicated that both BBR treatment and miR-214-3p mimic could inhibit the proliferation of MCF-7 and MDA-MB-231 cells. Scratch wound healing assay (Figure 3) and gelatin-based 3D conformation assay (Figure 4) suggested that both BBR treatment and miR-214-3p mimic could repress the migration capacities of MCF-7 and MDA-MB-231 cells. It was observed that both BBR and miR-214-3p mimic prevented the invasion capacities of MCF-7 and MDA-MB-231 cells by transwell invasion assay (Figure 5). However, miR-214-3p inhibitor counteracted all these suppressions of BBR treatment (Figures 2–5).

### 3.3. BBR Upregulates miR-214-3p Expression to Induce Cell Apoptosis and G2/M Arrest

**Figure 6:**
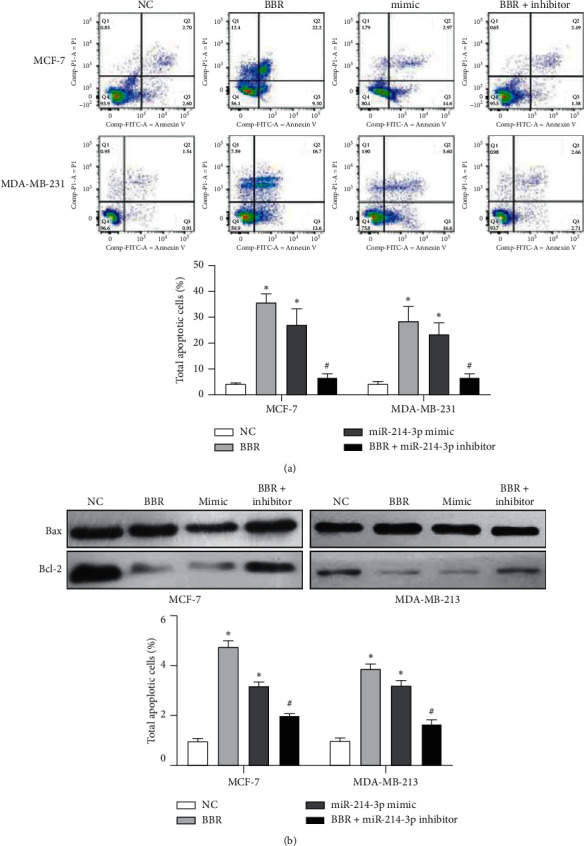
Cell apoptosis assays. (a) The total apoptotic cells and representative scatter plots. After BBR and miR-214-3p mimic administration, there were significant increases of 8.3-fold and 6.9-fold in the rates of total apoptotic cells in MCF-7 cells and 6.3-fold and 4.8-fold in MDA-MB-231 cells. (b) Western blot showed BBR and miR-214-3p mimic treatment increased the ratio of Bax/Bcl-2 by 4.8-fold and 3.1-fold in MCF-7 cells and by 3.9-fold and 3.2-fold in MDA-MB-231 cells. ^*∗*^*p* < 0.05 vs. NC group. ^#^*p* < 0.05 vs. BBR group.

**Figure 7:**
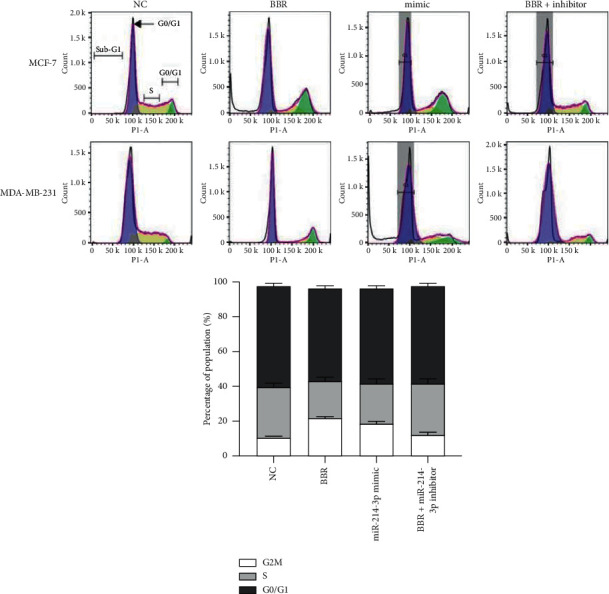
Cell cycle analyses. BBR and miR-214-3p mimic administration could increase the percentage of G2/M cells by 20% and 17% in MCF-7 cells and by 13% and 9% in MDA-MB-231 cells, respectively. ^*∗*^*p* < 0.05 vs. NC group. ^#^*p* < 0.05 vs. BBR group.

After BBR and miR-214-3p mimic administration, there were significant increases of 8.3-fold and 6.9-fold in the rates of total apoptotic cells in MCF-7 cells and 6.3-fold and 4.8-fold in MDA-MB-231 cells, respectively (Figure 6(a)). BBR and miR-214-3p mimic treatment increased the ratio of Bax/Bcl-2 by 4.8-fold and 3.1-fold in MCF-7 cells and by 3.9-fold and 3.2-fold in MDA-MB-231 cells, respectively (Figure 6(b)). All these indicated that both BBR and miR-214-3p mimic induce apoptosis of breast cancer cells. Cell cycle analysis was employed to compare the levels of cell combination dose induced by cell loss. The results indicated that BBR and miR-214-3p mimic administration could increase the percentage of G2/M cells by 20% and 17% in MCF-7 cells and by 13% and 9% in MDA-MB-231 cells, respectively (Figure 7). However, miR-214-3p inhibitor counteracted all these stimulations of BBR treatment (Figures 6 and 7).

### 3.4. BBR Promotes miR-214-3p Expression and Represses Protein Expression of Its Targets SCT

**Figure 8:**
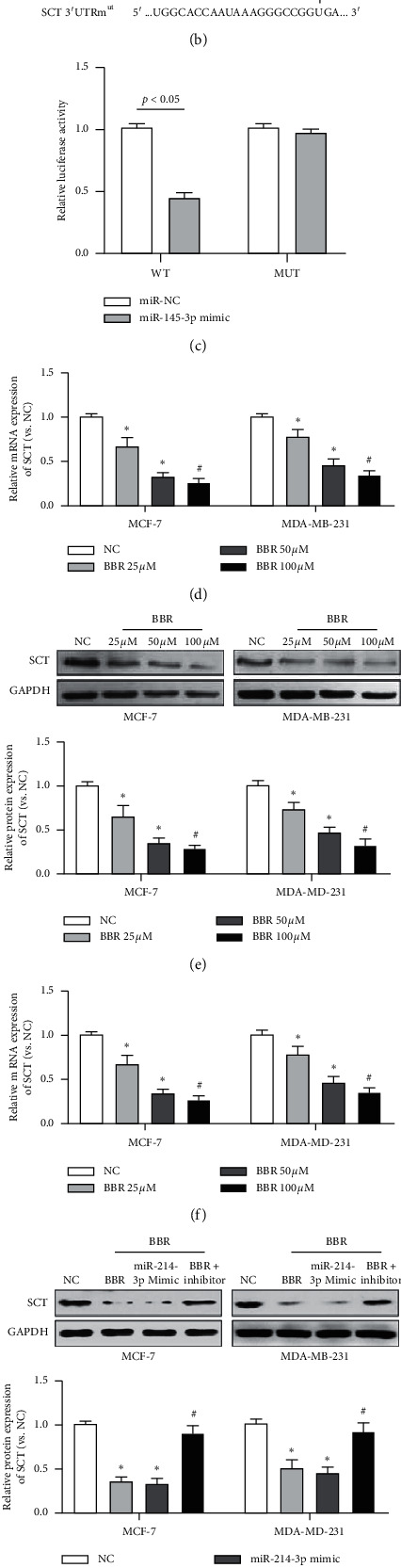
(a and b) Targetscan7.2 and miRwalk bioinformatics analysis identified that SCT and miR-214-3p had targeted binding sites. (c) Activity detection of dual-luciferase reporter gene. (d and e) BBR massively and dose-dependently reduced the mRNA expression and protein levels of SCT in both MCF-7 and MDA-231 cells. (f and g) Both miR-214-3p mimic and BBR decreased the mRNA expression and protein levels of SCT, while miR-214-3p inhibitor weakened these reductions. ^*∗*^*p* < 0.05 vs. NC group. ^#^*p* < 0.05 vs. BBR group.

Based on the Targetscan7.2 and miRwalk bioinformatics analysis, it was identified that SCT and miR-214-3p had targeted binding sites (Figures 8(a) and 8(b)). So, we first verified it by dual-luciferase reporter. Activity of luciferase in miR-214-3p mimic group inpsiCHECK-2-SCT-WT was obviously lower than that of independent sequence group (*p* < 0.05), while activity of luciferase in miR-214-3p mimic group in psiCHECK-2-SCT-MUT group was not significantly different from that of independent sequence group (*p* > 0.05) (Figure 8(c)). Then, to ensure the assumption that BBR could promote miR-214-3p expression and suppress the protein expression of its targets SCT, we further confirmed that BBR could massively and dose-dependently reduce the mRNA expression and protein levels of SCT in both MCF-7 and MDA-231 cells (*p* < 0.05) (Figures 8(d) and 8(e)). Next, we testified that both miR-214-3p mimic and BBR could decrease the mRNA expression and protein levels of SCT, while miR-214-3p inhibitor weakened these reductions (Figures 8(f) and 8(g)). These results indicated that BBR promoted miR-214-3p expression and repressed the protein expression of its targets SCT.

## 4. Discussion

BBR is a natural alkaloid mainly found in the famous Chinese herb Coptidis Rhizoma. In the beginning, BBR was used in treating diarrhea and gastroenteritis [37]. In subsequent studies, BBR was proved to possess other properties, for instance, antibiosis, cardioprotection, glucose regulation, and antineoplastic activity [12, 13, 35, 38]. In this study, it was found that BBR obviously suppressed the abilities of growth and invasiveness in both MCF-7 and MDA-MB-231 cells. These results were consistent with previous studies [39, 40]. Kim et al. found that berberine could efficiently inhibit growth by inducing cell cycle arrest in anoikis-resistant MCF-7 and MDA-MB-231 cells [39]. It was also indicated that the growth inhibitory effects of berberine treatment on MCF-7 cells might be partly due to the effects on side population cells and ABCG2 expression [40]. Further analysis of these phenotypes is essential for understanding the effect of berberine on anoikis-resistant breast cancer cells. Nonetheless, the antitumor mechanism of BBR in breast cancer cells still remains ambiguous.

In our investigation, the focus was to seek the key ncRNA and its target pathway in the antibreast cancer mechanism of BBR. We first listed a panel of 18 miRNAs [6–8, 16–30] and 9 lncRNAs [9, 10, 15, 29–34] that were previously reported to be related with breast cancer, and we found that BBR illustrated a dosage-dependent pattern in the stimulation to miR-214-3p in both MCF-7 and MDA-MB-231 cells. miRNA is one of the important regulators that act as a posttranscriptional suppression officer. Abnormal levels of miRNA could result in a diversity of regulation in diverse cellular pathways. There is a strong proof that some crucial miRNAs are involved in the progress of breast cancer [41]. To validate the role of miR-214-3p in the suppression of BBR to breast cancer cells, we performed a rescue test and observed that both BBR and miR-214-3p mimic could repress the abilities of growth, invasiveness, and migration in MCF-7 and MDA-MB-231 cells, while miR-214-3p inhibitor counteracted these suppression. We also found that BBR upregulated miR-214-3p expression to induce cell apoptosis and G2/M arrest. These results indicated that BBR presented anticancer effects through miR-214-3p. Another study showed that lncRNATSLNC8 inhibited miR-214-3p/FOXP2 axis to suppress the proliferation and G1/S phase transition of breast cancer cells [42]. It has been reported that the expression of miR-214-3p is correlated with the proliferation and apoptosis of breast cancer cells [21] and acts as a breast tumor suppressor through the regulation of EMT [43]. In the fields of breast cancer, these studies have established that miR-214-3p had a vital role in the progress of breast cancer. Our results indicated that miR-214-3p was also the target of BBR to present anticancer effects.

Then, after using bioinformatics analysis and searching previous studies, we identified that SCT might be the downstream target of miR-214-3p. To confirm the assumption that BBR could promote miR-214-3p transcription and raise the suppression of its downstream target SCT, we first confirmed that BBR could dose-dependently reduce SCT in both levels of mRNA and protein. After that, we found that both miR-214-3p mimic and BBR repressed SCT mRNA and protein, while miR-214-3p inhibitor weakened these reductions. In physiological conditions, SCT binds to its receptor to mediate the effect of the gastrointestinal hormone on digestion and water homeostasis. The overexpression of SCT in MCF-7 cells led to an increase of the cell proliferation index and cellular migration [44]. The data of this study revealed the fact that miR-214-3p inversely acted on SCT in both MCF-7 and MDA-MB-231 cells. However, whether SCT is an oncogene or a tumor suppressor is still controversial. A number of studies hold the idea that it is an oncogene, as high expression was observed in breast cancer [44], whereas other studies indicated the downregulation of the gene in colorectal cancer [45] and prostate cancer [46]. SCT acts as a gene with double-edge sword activities, which possesses both oncogenic and tumor-suppressive effects. It plays a tumor-suppressive role in normal cells and a proliferation and migration stimulating role in cancer cells. It has been reported that SCT suppresses the proliferation of normal breast cells, while the gene stimulates the proliferation and migration of cancer cells [44].

In conclusion, BBR is indicative of the suppression to MCF-7 and MDA-MB-231 breast cancer cells by upregulating the expression of miR-214-3p and increasing its inhibition to SCT. The miR-214-3p/SCT axis is the therapeutic target in the mechanism of BBR to suppress breast cancer.

## Figures and Tables

**Table 1:** Primer sequences.

	Forward	Reverse
miR-101	5′-UACAGUACUGUGAUAACUGAA-3′	5′-CAGUUAUCACAGUACUGUAUU-3′
miR-21	5′-CGCGCTAGCTTATCAGACTGA-3′	5′-GTGCAGGGTCCGAGGT-3′
miR-155-3p	5′-CCACAGGTGATGGGCAGAAT-3′	5′-TTCCTGTGGGGGATCGGTAT-3′
miR-381	5′-CCAGAUCGUAAGUGGUACCGUU-3′	5′-CUCUACACCGAACUAUAUCAGU-3′
miR-216b-5p	5′-CCTGGCGTCGTGATTAGTG-3′	5′-TCAGTCCTGTCCATAATTAGCC-3′
miR-205-3p	5-GAGGATCCCCGGGTACCGGTAGGCCTTT‐3′	5′‐CACACATTCCACAGGCTGCTACGGTGGTGGCGT‐3′
miR-200c	5′-GGGAACACACCTGGTTAAC-3′	5′-CAGTGCGTGTCGTGGAGT-3′
miR-188-5p	5′-GCG CAT CCC TTG CAT GGT-3′	5′-AGT GCA GGGTCCGAG GTATT-3′
miR-214-3p	5′-GCACAGCAGGCACAGACA-3′	5′-CAGAGCAGGGTCAGCGGTA-3′
miR-616	5′-ACACTC CAGCTGGGAGTCATTGGAGGGTTT-3′	5′-TGGTGTCGTGGAGTCG -3′
miR-129-5p	5′-AATCTAGAA CCCTGCCTGTGGTCCTGA-3′	5′-AACTCTAGA AGAGAGTCCCTAGT-3′
miR-26a-5p	5′-AGAAGATGGCA GCAAGAGCG-3′	5′-TCAAGTCAGGCTGAGATGCTAGT-3′
miR-203a	5′-TTGGATCACAGCGATACAAACTT-3′	5′-AGCGCACGCCAATAAAGACAT-3′
miR-7	5′-AAAAGAACACGTGGAAGGATAG-3′	5′-CGCCTAACGTACCGCGAATTT-3′
miR-211-5p	5′-CCCTTTGTCATCCTTCGCCT-3′	5′-GCGAGCACAGAATTAATACGACTC-3′
miR-138-5p	5′-TGCAAT GGGTTTGGCGTAGAAC-3′	5′-CCAGTGCCG CAGGGTAGGT-3′
miR-186-5p	5′‐CCCGA TAAAGCTAGATAACC‐3′	5′‐CAGTGCGT GTCGTGGAGT‐3′
LINC02582	5ʹ-ATCAACAGCCAACAAATACC-3ʹ	5ʹ-TTCTTATCACCGTCACCCT-3ʹ
SNHG3	5ʹ-TTCCGGGCGTTACTTAAGG-3ʹ	5ʹ-GGTCAAGAACAAGCACACCAA-3ʹ
AC073284.4	5′-TCATGGCTCACTGCAGCCTC-3′	5′-TGGGAGGCCAAGGTGACAGA-3′
H19	5′-ATCGGTGCCTCAGCGTTCGG-3′	5′-CTGTCCTCGCCGTCACACCG-3
Lnc101069	5′-GCTTAGAAATTTCTTCCACCTG-3′	5′-CTGCCCTAGCGATTTGTGAA-3′
DRHC	5′-CAGTGGGGAACTCTGACT CG-3′	5′-GTGCCTGGTGCT CTCTTACC-3′
RUNXOR	5′-ATGTTTAGTATTTTAAATGATGGGATT-3′	5′-ACCTACCCTCCCCCAAACTATAC-3′
LINC01287	5′-CCGCATCCAAACCTACATACTAACCC-3′	5′-CGACCGAAAAAATTCCATTCCCTCAA-3′
SNHG6	5′-TTGGGATGTTGATAGTTTTAGATGGAGGT-3′	5′-AATAAATCCATCCCTCATAACRA-3′
GAPDH	5′-GGGAGCCAAAAGGGTCAT-3′	5′-GAGTCCTTCCACGATACCAA-3′

## Data Availability

The datasets used and/or analyzed in the present study are available from the corresponding author upon reasonable request.
